# Meetings of Societies

**Published:** 1911-03

**Authors:** 


### MEETINGS OF SOCIETIES.

### Bristol Medico-Chirurgical Society.

*December 14th*, 1910.

Dr. Bertram M. H. Rogers, President, in the Chair.

Captain F. P. Mackie, I.M.S., F.R.C.S., Member of the Royal Society's Sleeping Sickness Commission, gave an address, illustrated by microscope, lantern and kinematograph, on **Trypanosomiasis in man and animals.** The subject was discussed under the following headings :—

(1) Introductory remarks.
(2) Scope of the inquiry of the Commission.
(3) Brief outline of sleeping sickness.
(4) Trypanosomiasis : its general characters and its special characters as studied in Uganda.
(5) Methods of differentiating trypanosomes.
(6) Mode of spread of the disease.
(7) The tsetse fly, its anatomy and natural parasites.
(8) The fate of the parasite of sleeping sickness in the fly.
(9) Mechanical transmission of the parasite.
(10) Prolonged infectivity of the lake shore after removal of the native inhabitants.
(11) Question of the existence of a reservoir in some host that harbours the parasite without developing the disease.
(12) The trypanosome in man.
(13) The effect of the trypanosome infection and nervous symptoms.
(14) The prevention and cure of sleeping sickness.

Kinematograph films, prepared and exhibited by Pathé Frères, London and Paris, showed "living pictures" of T. Gambiense and T. Lewisii in the blood, and the spirochete of relapsing fever.

A cordial vote of thanks, proposed by the PRESIDENT, seconded by Dr. D. S. DAVIES, was given to Captain Mackie for his most interesting and instructive address.

---

*January 11th*, 1911.

Dr. BERTRAM M. H. ROGERS, President, in the Chair.

Dr. WALKER HALL and Dr. G. SCOTT WILLIAMSON described **The technique of a test in which ferment action was used as a means of diagnosing gastric cancer.**—They had obtained good results in cases where the clinical diagnosis was confirmed at operation or autopsy. Precautions were necessary with regard to the presence of bile, blood and duodenal contents. The polypeptide used as an indicator was glycyl-tryptophane. It may be obtained from Kalle, of Biebrich. Drs. Walker Hall and Williamson also gave an account of some observations they had made upon the dipeptide splitting action of blood and pathological fluids. Using glycyl-tryptophane for this purpose, they found that a cleavage took place when this substance was incubated with blood and tissue fluids. The action was associated with the globulin reaction of the pathological material, and with regard to time and reaction possessed characters akin to ferments. The results so far accumulated suggested that future work might reveal methods by means of which the findings might be applied practically.

Dr. G. PARKER gave an **Account of an outbreak of infantile paralysis,** numbering thirty-seven cases, which occurred **in Bristol** from June, 1909, to January, 1910. He referred to the extraordinary epidemics in recent years both in Europe and America, and suggested that this group of cases represented much more than the ordinary sporadic crop. Thirty-two of them appeared in the summer, or at least before the end of October. There were nearly twice as many males as females, and the average age, excluding three adolescents, was two years. Two deaths were known to have occurred, and the great majority of the children were crippled for life. The manner of transmission of the infection is very obscure. Well-marked instances of contagion, and of conveyance by healthy carriers, have been frequently recorded, while Flexner and others have reproduced the disease again and again by injecting an emulsion of the spinal cord of an infected animal. In Bristol there was little evidence of direct contagion, except that two children in one family were attacked about the same time. The initial symptoms frequently included fever, rheumatic pains, pulmonary and throat troubles, though in some cases all of these were absent.

---

*February 8th*, 1911.

Dr. BERTRAM M. H. ROGERS, President, in the Chair.

Dr. A. J. WRIGHT showed a number of specimens to illustrate points in the **Surgical anatomy of the mastoid process.** He maintained that his specimens supported the contention that the so-called sclerosis of the mastoid was a natural condition due to persistence of the type of mastoid process met with naturally in all cases at five years of age. The density of the outer wall of the antrum in this condition of the bone disposed to intracranial extension of inflammatory processes originating there. In the only two specimens obtained from fatal cases of such extension the bones showed this density of the outer antral wall. In this type of bone the lateral sinus was often situated far forwards, making access to the antrum by operation difficult.

Dr. W. KENNETH WILLS read a paper on **An anomalous form of ringworm.** The patient, a child of two years of age, was troubled with an obstinate vesicular eruption on the foot, which was found to be due to ringworm. The history of the discovery of the tricophyton by Sabouraud in this kind of affection was described, and allusion made to a visit paid by the speaker to the clinic of that dermatologist. Emphasis was laid upon the importance of microscopical search for the tricophyton in the macerated epithelium and vesicular fluid in certain cases of obstinate eruption on the skin.—Dr. NIXON discussed the paper, and gave many interesting details of cases of persistent tricophyton infection in schools and colleges. The treatment had to be severe to be of any use, but at best was not very satisfactory.

Mr. W. ROGER WILLIAMS read a paper on **Tumour of a dog's tail,** and showed the specimen. It was a subcutaneous tumour in the tail of a fox-terrier about fourteen years old. It had an innocent appearance, was firm and encapsuled, and solid in texture, with a tendency to lobulation. The tumour had slowly increased from the size of a split pea when first detected to its present size (about that of a bantam's egg) in two and a half years. The animal was destroyed in the end because it had spasmodic fits of coughing for some months. A few small nodules were found on the surface of the spleen. The section of the tumour under the microscope showed a fibro-leiomyomatous structure. Tumours of this kind had been found in humanity, and were generally believed to originate in connection with arrector pili muscles. Similar tumour tissue was found in the spleen and in an enlarged gland removed from near the lower end of the œsophagus of the dog.—Mr. C. A. MORTON demonstrated (1) **A specimen of ruptured tubal gestation** removed eighteen months after operation for similar condition on the other side; (2) **A specimen of malignant ovarian tumour** removed from a girl aged 12.

J. LACY FIRTH.
J. A. NIXON, *Hon. Sec.*

